# Stereo Matching by Filtering-Based Disparity Propagation

**DOI:** 10.1371/journal.pone.0162939

**Published:** 2016-09-14

**Authors:** Xingzheng Wang, Yushi Tian, Haoqian Wang, Yongbing Zhang

**Affiliations:** 1 Shenzhen Key Laboratory of Broadband Network & Multimedia Graduate School at Shenzhen, Tsinghua University, Shenzhen, China; 2 Shenzhen Institute of Future Media Technology, Shenzhen, China; Tianjin University, CHINA

## Abstract

Stereo matching is essential and fundamental in computer vision tasks. In this paper, a novel stereo matching algorithm based on disparity propagation using edge-aware filtering is proposed. By extracting disparity subsets for reliable points and customizing the cost volume, the initial disparity map is refined through filtering-based disparity propagation. Then, an edge-aware filter with low computational complexity is adopted to formulate the cost column, which makes the proposed method independent on the local window size. Experimental results demonstrate the effectiveness of the proposed scheme. Bad pixels in our output disparity map are considerably decreased. The proposed method greatly outperforms the adaptive support-weight approach and other conditional window-based local stereo matching algorithms.

## Introduction

**Fig 1:**
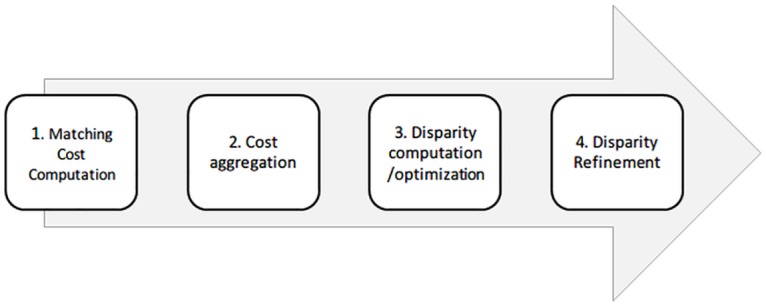
Four-step framework of Stereo matching.

Stereo matching solves the correspondence problem between stereo image pairs, which for a long time has been one of the most fundamental and challenging computer vision tasks. As designed by Scharstein and Szeliski [1], and which is widely acknowledged by later researches, a four-step framework of Stereo matching (as Fig 1 shows): matching cost computation, cost aggregation, disparity computation and disparity refinement may generate the dense and two-frame stereo problem. Most existing algorithms, including local, global as well as semi-global ones, perform all or some of these four steps.

Local stereo matching algorithms usually perform cost computation and aggregation by a simple winner-take-all (WTA) strategy. In contrast, global approaches transform the problem to an energy-minimization model, which formulate a global optimization function composed of a data term and a smoothness term, and perform global disparity optimization [2–9] by dynamic programming (DP), graph cuts (GC) [10,11] or belief propagation (BP) [12–15]. Although high accuracy can be achieved for disparity estimation through global optimization, the large computation and time cost also limits its implementation in real-time applications. Moreover, segment-based approaches utilize plane fitting from the initial disparities on each segment, based on the assumption that disparities vary smoothly and continuously within each homogeneous color segment. The result can be further improved by a global optimization model to find optimal parameters of disparity plane, as the labeling space is relatively small. Recently the concept of semi-global stereo matching is also proposed based on a recognition stage that the support pixels for cost aggregation should be selected from the whole image and not restricted in a local matching window. Representatively, Hirschmuller introduced the mutual information to compute pixel wise matching cost and aggregate the costs along multiple paths that end in the current pixel [16]. Yang built a graph using all image pixels as nodes and compute the matching cost adaptively based on pixel similarity on a minimum spanning tree (MST) [17]. Matching cost aggregation plays a critical role to reduce the mismatching rate when using the per-pixel matching function. For most local stereo matching algorithms this is achieved by summing up or averaging the matching costs in a surrounding window centered by the current pixel. Using adaptive support-weight [18,19] for neighbor pixels will take edges or textures into account and bring better performance by adapting big date processing technologies [20–24].

In this paper a filtering-based stereo matching algorithm is proposed. Based on the perception that aggregating matching costs in a rectangular window is equivalent to filter the cost volume, we conducted the adaptive support-weight cost aggregation through filtering. Particularly we used the edge-aware guided filter which has linear time complexity with respect to the image size, it would reduce the computational complexity and greatly save the running time. Then the reliable and unreliable points were obtained through crosschecking of two rough-estimated disparity maps. Lastly, the high confidence disparity estimates are propagated from reliable points to unreliable ones by filtering a customized cost volume. A feedback-based optimization can also be achieved by integrating the initial disparity map into the guide image and re-execute the filtering progress. The main novelty of the proposed method is the utilize of reliable points, by propagating of these point, high performance of stereo matching would be achieved.

The rest of this paper is organized as follows. Section 2 explains the idea that matching cost aggregation is equivalent to the cost volume filtering. In Section 3 the concept of reliable point and disparity subset is first introduced, then we show how to customize a new cost volume to propagate the useful information from reliable points to unreliable ones by applying edge-aware filtering. Experimental results are demonstrated in Section 4, along with some analysis and evaluation. Finally, we conclude our paper and discuss the future work in Section 5.

## Cost Aggregation by Cost Volume Filtering

The robust dissimilarity measure taking both SAD and gradient into account [13] is used as the pixel-wise matching cost function. Then a cost aggregation procedure which sums up costs in a window is usually implemented for local stereo matching. In this way, cost aggregation is equivalent to applying filtering on the initial cost volume. Furthermore, the adaptive support-weight can be achieved by an edge-aware filter such as bilateral filter. For non-linear filtering each output pixel is calculated in O(*r*^2^) if the kernel size is r * r, thus the computational complexity shoots up as the kernel size increases. The so-called O(1) or constant time bilateral filter [25,26], meaning the computational complexity is invariant to the kernel size, is designed for fast implementation.

In this paper we adopt the guided filter proposed by *He et al*. [27] to conduct the cost volume filtering. The guided filter is based on a local linear model, assuming that in a local window the filtering output *q* can be expressed as a linear transform of the guided image I:

$$q_{i}=a_{k}^{T}I_{i}+b_{k},\;\forall i\in \omega_{k}$$ (1)

Where (*a*_*k*_, *b*_*k*_) are linear coefficients which are constant in *ω*_*k*_, Considering the constraints from the filtering input *p* the linear coefficients can be derived as:

$$\begin{array}{l} {\text{a}}_{k}={\left(\Sigma_{k}+\varepsilon U\right)}^{-1}(\frac{1}{\left|w\right|}\sum_{i\in w_{k}}{\text{I}}_{i}p_{i}-\mu_{k}{\bar{p}}_{k})\\ b_{k}={\bar{p}}_{k}-{\text{a}}_{k}^{T}\mu_{k} \end{array}$$ (2)

Here, Σ_*k*_ is the 3*3 covariance matrix of I in *ω*_*k*_, and U is a 3*3 identity matrix, *μ*_*k*_ is the mean of image I. Then compute the output q using:

$$q_{i}={\bar{a}}_{i}^{T}I_{i}+{\bar{b}}_{i}$$ (3)

The guided filter is also edge-aware like bilateral filter, yet has better performance near edge locations. Another advantage of guided filtering is that the time complexity is only O(1) for each pixel and O(*N*) for an image of *N* pixels. In contrast, traditional edge-aware filters such as bilateral filter has O(*r*^2^) time complexity for each pixel when the local filter window has size of *r* * *r*, thus for the whole image it’s O(*Nr*^2^) time. As the size of filter kernel increases, the time cost for such filters increases rapidly as well. Although the guided filter follows the local linear assumption, its computation complexity is unrelated to the local window size. This property makes it more practical for cost volume filtering.

When performing guided filtering on the cost volume C, the input image to be filtered is a certain slice at disparity candidate d in the cost volume, and the input color image of reference view is used as the guide image. An initial disparity map can be generated using the winner-take-all (WTA) strategy based on the filtered cost volume.

## Disparity Propagation of Reliable Points

The left-right consistency check is widely used to verify the accuracy of the disparity estimation for each pixel. In this work pixels that pass the cross-check are marked as reliable points, and accordingly the others are unreliable ones. It is reasonable that the information of reliable points in the cost volume has higher confidence and should do favor to other pixels.

### A. Building Disparity Subsets

**Fig 2:**
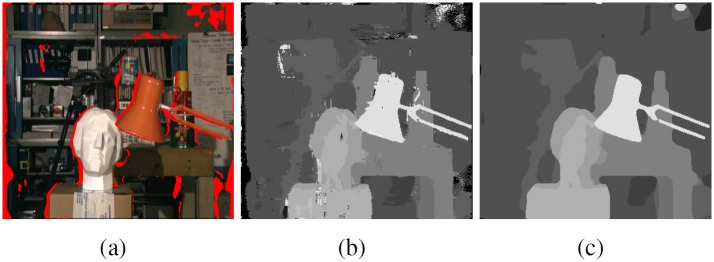
The “Tsukuba” test image. (a) Unreliable regions marked in red. (b) Initial disparity map. (c) Refined disparity map after filtering-based disparity propagation.

Denote the full disparity range as *D*_*full*_, a small subset *D*_*sub*_(*p*) is built for each reliable pixel *p* containing a couple of disparity candidates corresponding to the |*D*_*sub*_(*p*)| minimal matching costs in the cost volume. The size of the subset should be small as |*D*_*sub*_(*p*)| ≪ |*D*|. The extracted disparity values have the highest confidence among all candidates for the current pixel. Disparities out of this subset will be punished when filtering the cost volume. Fig 2 (a) shows the result of the cross-check based on the initial disparity map in (b). The unreliable pixels failing the cross-check are marked in red. Computing disparities in these red regions has to consult to the useful information from nearby reliable points.

### B. Disparity Propagation Base on Customized Cost Volume

We customize a new cost volume based on the initial disparity map and the disparity subsets of reliable points:

$$C_{d}^{\prime }(p)=\{\begin{array}{cc} \left|d-D(p)\right|+\delta (p,d), & p\in P_{reliable}\\ 0, & else \end{array}$$ (4)

Where *P*_*reliable*_ is the set of reliable pixels. D is the dense disparity map, *δ*(*p*, *d*) is the penalty factor. The costs for unreliable points are set to 0 hence to eliminate the negative impact of wrong estimates, while for reliable points, disparities closer to the initial estimate *D*(*p*) still have smaller values in the new cost volume. *δ*(*p*, *d*) is the penalty term to avoid being too far away from the disparity subset in the new estimation.

$$\delta (p,d)=2-\text{exp}(-\lambda_{1}\underset{\begin{array}{c} d_{s}\in D_{sub}(p)\\ \left|d-d_{s}\right|\leq 1 \end{array}}{\sum }\left|d-d_{s}\right|)-\text{exp}(-\lambda_{2}\underset{\begin{array}{c} d_{s}\in D_{sub}(p)\\ \left|d-d_{s}\right|>1 \end{array}}{\sum }\left|d-d_{s}\right|)$$ (5)

Where *λ*_1_ < *λ*_2_ indicating that disparities far from the subset *D*_*sub*_(*p*) will lead to larger penalty. However, the penalty is limited as we use the exponential function.

Once again we perform the WTA optimization at each pixel and get a refined disparity map. A constant-time median filter is then applied as post-processing to fill holes and remove peaks.

### C. Integrating Disparity into Guided Filtering

The RGB image of the reference view is used as the guide image when filtering the cost volume. Notice that before the new volume is generated an initial disparity map is available. The estimated disparity can feed back to the filtering procedure as an extra channel of the guide image, and bear a hand in filtering the customized cost volume. We integrate the disparity channel into the RGB reference image and form a new RGB-D guide image. For guided filtering this means simply replace the three-dimensional vector *I*_*i*_ in Eqs (2) and (4) with a four-dimensional one. Then disparity propagation using the new guide image and the customized cost volume will lead to refinement and optimization of the disparity map.

## Experimental Results

**Fig 3:**
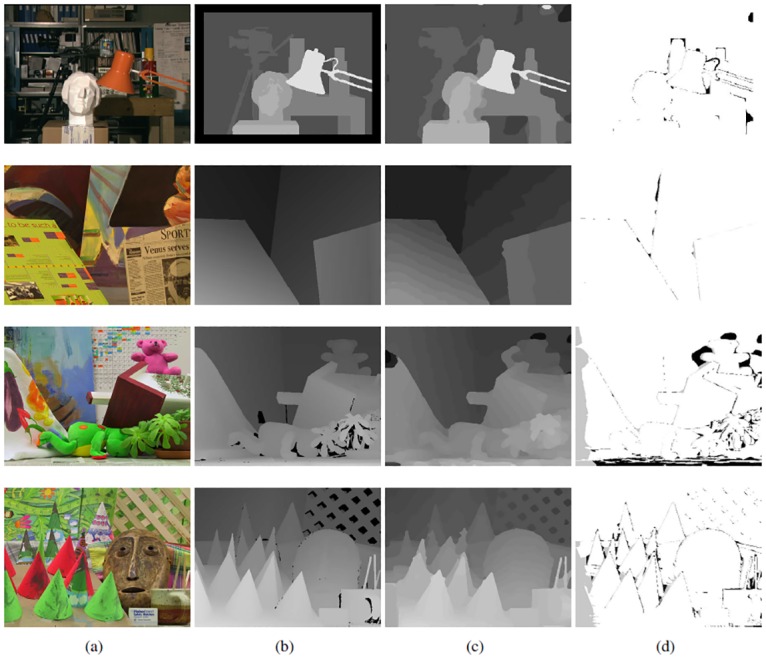
Results of the proposed algorithm. (a) Left view of the input image pair. (b) Ground truth disparity map. (c) Resulting disparity map using our method. (d) Bad pixels with error lager than 1.0.

Our method is evaluated on the standard Middlebury benchmark. All the experiments run on a PC platform equipped with Intel Core i5 CPU and 4GB memory. The size of the disparity subset *D*_*sub*_ = 2, and parameters *λ*_1_ and *λ*_2_ are set to 0.04 and 1.2, respectively. Fig 3 shows the left view of the four test image pairs “Tsukuba”, “Venus”, “Teddy”, “Cones” and their corresponding ground truth disparity. The third column shows the disparity maps generated using the proposed algorithm. Bad pixels with absolute disparity error larger than 1.0 are marked out in the last column. It can be observed that the proposed algorithm recovers satisfactory disparity maps even for complicated scenes. Most of the bad pixels lie near edges where occlusion often occurs and is challenging for all stereo matching algorithms.

Table 1 gives the quantitative evaluation indicators of our results with error threshold = 1, along with results of some other representative algorithms such as adaptive support-weight approach and global optimization algorithms using Graph Cuts or Belief Propagation. Our method outperforms the others with respect to the average percent of bad pixels, especially for the “Cones” image pair on which our method is in the top 3 considering all 3 indicators including bad pixels in regions near discontinuities, non-occluded regions and all regions. For the other test image pairs our results still occupy the advanced level among all algorithms listed here. The proposed guided filtering-based disparity propagation outperforms the adaptive-weight approach for all test images except for “Tsukuba”. Moreover, out algorithm maintain the edge-aware property just as adaptive-weight yet has obvious advantage on computation complexity, as guided filtering is O(1) time for each pixel and regardless of the filter kernel size. This makes it easier to use bigger local window size when handling images of large size without concern of sharp rise of the time cost.

**Table 1:** Middlebury error rates of different algorithms (Error Threshold = 1).

Algorithm	Tsukuba			Venus			Teddy			Cones			Bad pixels(%)
	nonoc	all	disc	nonoc	all	disc	nonoc	all	disc	nonoc	all	disc	
**Ours**	1.73	2.14	7.54	0.33	0.75	4.5	7.39	13.1	17.9	2.57	8.52	7.56	6.17
**CostAggr+occ**	1.38	1.96	7.14	0.44	1.13	4.87	6.8	11.9	17.3	3.6	8.57	9.36	6.2
**MVSegBP** [7]	1.06	2.78	5.57	0.2	0.61	2.02	6.53	11.3	14.8	5.29	11.3	14.5	6.34
**RandomVote**	4.85	5.54	17.7	0.13	0.45	1.86	5.4	9.54	14.8	2.62	7.93	7.54	6.53
**GradAdaptWgt** [11]	2.26	2.63	8.99	0.99	1.39	4.92	8	13.1	18.6	2.61	7.67	7.43	6.55
**AdaptWeight** [10]	1.38	1.85	6.9	0.71	1.19	6.13	7.88	13.3	18.6	3.97	9.79	8.26	6.67
**EnhancedBP** [6]	0.94	1.74	5.05	0.35	0.86	4.34	8.11	13.3	18.5	5.09	11.1	11	6.69
**SemiGlob** [8]	3.26	3.96	12.8	1	1.57	11.3	6.02	12.2	16.3	3.06	9.75	8.9	7.5
**RealtimeBP**	1.49	3.4	7.87	0.77	1.9	9	8.72	13.2	17.2	4.61	11.6	12.4	7.69
**GC+occ** [3]	1.19	2.01	6.24	1.64	2.19	6.75	11.2	17.4	19.8	5.36	12.4	13	8.26

The running time of proposed method is as Table 2 shows, All the experiments run on a Laptop T450s equipped with Intel Core i5-5200U CPU and 4GB memory.

**Table 2:** Running time of proposed method.

Image	Running time (s)
**cones**	11.591
**teddy**	12.044
**venus**	2.618
**tsukuba**	4.570

We also test various combinations of parameters including the size of disparity subsets as well as *λ*_1_, *λ*_2_ in the penalty term. Table 3 gives the average percent of bad pixels on the above 4 test image pairs when using various combinations of parameters, which is denoted in the form of (|*D*_*sub*_|, *λ*_1_, *λ*_2_). Our experience suggests that the size of disparity subsets |*D*_*sub*_| in the range [2,3], and [0.02,1.0], [0.8,2.0] for *λ*_1_, *λ*_2_ respectively can achieve approximately optimal results. The selection of parameters is based on the experimental results on the test images, as listed in Table 3. Empirically the size of disparity subsets is set to be small, for the initial disparity value of a reliable points is regarded to be of relatively high confidence, thus large penalty should be given to avoid large deviation from the subsets.

**Table 3:** Error rates for various parameters (|*D*_*sub*_|, *λ*_1_, *λ*_2_).

Parameters	Bad pixels(%)
**(1,0.04,1.2)**	6.25
**(2,0.04,1.2)**	6.17
**(2,0.04,0.8)**	6.18
**(2,0.10,1.2)**	6.18
**(3,0.04,1.2)**	6.31
**(4,0.04,1.2)**	6.35

## Conclusions

This paper proposes a stereo matching algorithm based on disparity propagation using edge-aware filtering. By extracting disparity subsets for reliable pixels and define a new cost volume accordingly, mismatches in the initial disparity map are corrected through disparity propagation from nearby reliable points. The guided filtering is integrated to conduct the propagation in O(1) time, which shows great advantage compared to traditional window-based cost aggregation methods. Future work will focus on how to customize a more reasonable cost volume which is essential to the disparity propagation. Occlusion handling and post processing of the disparity map also remain to improve.
